# Temporal trends in inequalities of the burden of HIV/AIDS across 186 countries and territories

**DOI:** 10.1186/s12889-023-15873-8

**Published:** 2023-05-26

**Authors:** Penghong Deng, Mingsheng Chen, Lei Si

**Affiliations:** 1 School of Health Policy & Management, Nanjing Medical University, Jiangning District, 211166 Nanjing, China; 2 Center for Global Health, Nanjing Medical University, Nanjing, China; 3 School of Health Sciences, Western Sydney University, Campbelltown, NSW Australia; 4 Translational Health Research Institute, Western Sydney University, Penrith, NSW Australia

**Keywords:** HIV/AIDS, Global burden of disease, Age-standardized DALY rates, Socioeconomic inequalities, Concentration index

## Abstract

**Background:**

The Global Burden of Disease, Injuries, and Risk Factors Study (GBD) has reported that HIV/AIDS continues to take a disproportionate toll on global health. However, the trends in global inequality of HIV/AIDS burden have remained ambiguous over the past two decades. The objectives of our study were to assess the socioeconomic inequalities, and temporal trends of HIV/AIDS across 186 countries and territories from 2000 to 2019.

**Methods:**

We extracted data from the GBD 2019, and conducted a cross-national time-series analysis. Age-standardized disability-adjusted life-year (DALY) rates were used to measure the global burden of HIV/AIDS. Gross national income (GNI) per capita was used to approximate the national socioeconomic status. Linear regression analysis was conducted to investigate the relationship between age-standardized DALY rates due to HIV/AIDS and GNI per capita. The concentration curve and concentration index (CI) were generated to evaluate the cross-national socioeconomic inequality of HIV/AIDS burden. A joinpoint regression analysis was used to quantify the changes in trends in socioeconomic inequality of HIV/AIDS burden from 2000 to 2019.

**Results:**

A decrease in age-standardized DALY rates for HIV/AIDS occurred in 132 (71%) of 186 countries/territories from 2000 to 2019, of which 52 (39%) countries/territories achieved a decrease in DALYs of more than 50%, and 27 (52%) of the 52 were from sub-Saharan Africa. The concentration curves of the age-standardized DALY rates of HIV/AIDS were above the equality line from 2000 to 2019. The CI rose from − 0.4625 (95% confidence interval − 0.6220 to -0.2629) in 2000 to -0.4122 (95% confidence interval − 0.6008 to -0.2235) in 2019. A four-phase trend of changes in the CIs of age-standardized DALY rates for HIV/AIDS was observed across 2000 to 2019, with an average increase of 0.6% (95% confidence interval 0.4 to 0.8, P < 0.001).

**Conclusions:**

Globally, the burden of HIV/AIDS has decreased over the past two decades, accompanied by a trend of narrowing cross-country inequalities of HIV/AIDS burden. Moreover, the burden of HIV/AIDS continues to fall primarily in low-income countries.

**Supplementary Information:**

The online version contains supplementary material available at 10.1186/s12889-023-15873-8.

## Background

The human immunodeficiency virus (HIV) and acquired immunodeficiency syndrome (AIDS) are incurable diseases that have become epidemic and garnered widespread concern from the government and public since their emergence. They pose an unprecedented threat to global health [1]. Globally, approximately 38.4 (33.9–43.8) million people were living with HIV at the end of 2021, with 1.5 million new cases and 650,000 deaths from HIV-related causes in 2021 [2]. The epidemic primarily affects vulnerable populations living in certain low- and middle-income countries (LMICs), and some specific groups living in high-income countries [3]. Despite significant efforts by national and territorial initiatives to mitigate the adverse consequences of the epidemic on health [4, 5]. HIV/AIDS continues to impose a massive physical and economic burden on individuals, healthcare systems and society, especially in economically disadvantaged regions [6, 7].

HIV/AIDS burden is the result of a synergistic combination of the underdeveloped sociodemographic and poor access to and performance of healthcare systems. Recognizing the continuously increasing burden of HIV/AIDS, and to accelerate the termination of the pandemic worldwide, the United Nations Programme on HIV/AIDS (UNAIDS) has initiated the 95-95-95 program. The aim of this program is that by 2030, 95% of people living with HIV will be diagnosed, of whom 95% will receive combined antiretroviral treatment (ART, a critical approach for HIV suppression), and of these 95% will achieve viral suppression [8]. Between 2000 and 2015, motivated by the sustainable development goals (SDGs), over $500 billion was invested globally to control the HIV/AIDS epidemic, with the commitment to deliver equitable access to prevention, treatment services, and sustained care and safeguards for people living with HIV/AIDS [9]. These ambitious goals were predicated on extensive global collaboration, universal HIV education, and medical technology support. However, currently only 85% of people living with HIV identify their HIV status, 75% of whom receive ART, with lower figures in sub-Saharan Africa, where the burden of HIV/AIDS is the highest worldwide [2]. The past decades witnessed dramatic achievements in the global battle against pandemics, yet significant disparities existed between countries in their responses to the HIV pandemic, including HIV screening, care, clinical outcomes, economic protection, and human rights advocacy [1, 6]. Renewed focus on the current global burden of HIV/AIDS and related inequalities is essential for timely reorientation or enhancement of future strategies.

The Global Burden of Disease, Injuries, and Risk Factors Study (GBD) evaluated the burden of HIV/AIDS in 204 countries and territories worldwide [10], presenting a valuable opportunity to investigate the HIV/AIDS landscape. A study from GBD 2017 provided a comprehensive assessment of global, temporal, and national trends in HIV incidence, prevalence, and mortality, but failed to deliver a detailed analysis of changes in the overall burden of HIV/AIDS [8]. Another recent study showed a declining trend in the global burden of HIV/AIDS over the past 15 years, with an unfavorable trend in high and middle-income countries [11]. In addition, previous studies have revealed widespread inequalities in HIV infection, testing, care, and awareness by gender, race, and income, especially in impoverished areas [12–15]. An assessment of cross-country inequalities and trends in the global burden of HIV/AIDS has not yet been conducted.

In line with the UNAIDS goal of ending the HIV pandemic by 2030, measurement of socioeconomic inequalities of HIV/AIDS burden and the temporal trends in response to the growing populations living with HIV/AIDS is critical to ensure equity and effectiveness of HIV programs. This is particularly noteworthy since the Corona Virus Disease 2019 (COVID-19) has disrupted the critical services for people with HIV, increased susceptibility to HIV/AIDS, and fueled social inequality [16]. In the current study, we quantified the changes and socioeconomic inequalities in HIV/AIDS burden, and temporal trends in inequalities from 2000 to 2019 in 186 countries and territories worldwide.

## Methods

### Data sources

We conducted a cross-country time-series secondary analysis of data from GBD 2019. This data estimates the global burden of 369 diseases and injuries by age and sex group among 204 countries and territories from 1990 to 2019 [10]. The GBD provides comprehensive assessment and analysis of the most up-to-date epidemiological data available. It employs a variety of health-related metrics to assess the burden of disease, such as deaths and mortality, the number of cases and prevalence, years of life lost (YLLs) due to premature mortality, years lived with disability (YLDs), and disability-adjusted life years (DALYs) [10]. For our study, 2000–2019 data were obtained from the Global Health Data Exchange to assess trends in socioeconomic inequalities in the burden of HIV/AIDS. DALYs were used to measure the health burden of HIV/AIDS, calculated as the sum of the YLLs and YLDs, considering both the length and quality of life [10]. Given the rapid population growth and age composition changes, age-standardized DALY rates were used in our analysis [17]. Detailed methods used to compute age-standardized DALY rates have been described in other GBD publications [18].

### National socioeconomic status

Gross national income (GNI) per capita was used to represent the national socioeconomic status, indicating the overall economic state of a country and the general living standards of its citizens [19]. GNI measurement comprises all resident producers along with associated taxes not included within the output figures and net primary income from abroad [20]. GNI is distributed among the mid-year population of each country to yield the amount per capita [20]. In general, countries with a higher GNI per capita have a higher level of socioeconomic development than those with a lower per capita GNI. We carried out a logarithmic transformation of GNI per capita to interpret the nonlinearity due to marginal utility. GNI per capita data for each country are publicly available from the World Bank classification datasets [21].

### Measures of health inequality

The concentration curve and concentration index (CI) [22] were used to estimate the cross-national health inequality of HIV/AIDS burden from 2000 to 2019. The concentration curve displays the unequal distribution between the cumulative proportion of the age-standardized DALY rates and the cumulative country proportion ranked by GNI per capita. If the curve is above or below the line of equality (45-degree line), age-standardized DALY rates are more concentrated in countries with lower or higher socioeconomic status, respectively [22]. The CI is derived from the concentration curve, is defined as twice the area between the concentration curve and the 45-degree line, and quantifies the degree of socioeconomic inequality in HIV/AIDS burden [22]. CI ranges from − 1 to 1 and a positive or negative value implies that countries with higher or lower socioeconomic status, respectively, bear more burden of HIV/AIDS [22].

### Statistical analysis

A descriptive analysis was conducted to characterize the global burden of HIV/AIDS. We computed the change rate of age-standardized DALY rates from 2000 to 2019, and categorized countries into four groups: age-standardized DALY rates increased by 50% or above, increased by less than 50%, decreased by 50% or below, and decreased by more than 50%. Linear regression analysis was conducted to investigate the association between age-standardized DALY rates due to HIV/AIDS and GNI per capita. A joinpoint regression analysis [23] was used to explore significant changes in trends in socioeconomic inequality of HIV/AIDS burden from 2000 to 2019 by estimating the annual percentage change (APC) and average annual percentage change (AAPC) of the CIs. The APC shows the trends for each fixed interval, while the AAPC is a summary measure of the trend over the entire period, calculated as the weighted average of APC, with the weight equal to the length of APC intervals [24]. A significant change in the CIs over time was estimated by the permutation test, and the p-value and 95% confidence interval obtained from the test were estimated using the Monte Carlo method [23]. Bonferroni correction of p values was used for multiple comparisons. APC and AAPC values were considered statistically significant when they were unequal to zero at alpha of 0.05.

All statistical analyses were carried out using SPSS v26.0 (SPSS Inc., Chicago, IL, USA) and Stata v17.0 (Stata Corp., College Station, TX, USA). All joinpoint analyses were conducted using Joinpoint Statistical Software (Joinpoint Regression Program, Version 4.9.1.0-April 2022, National Cancer Institute, Bethesda, MD, USA; Statistical Methodology and Applications Branch, Surveillance Research Program, National Cancer Institute).

### Patient and public involvement

No patients or members of the public were directly involved in this study. There are no plans to involve patients or the public in the dissemination of results.

## Results

### Global burden of HIV/AIDS with age-standardized DALY rates

**Fig. 1:**
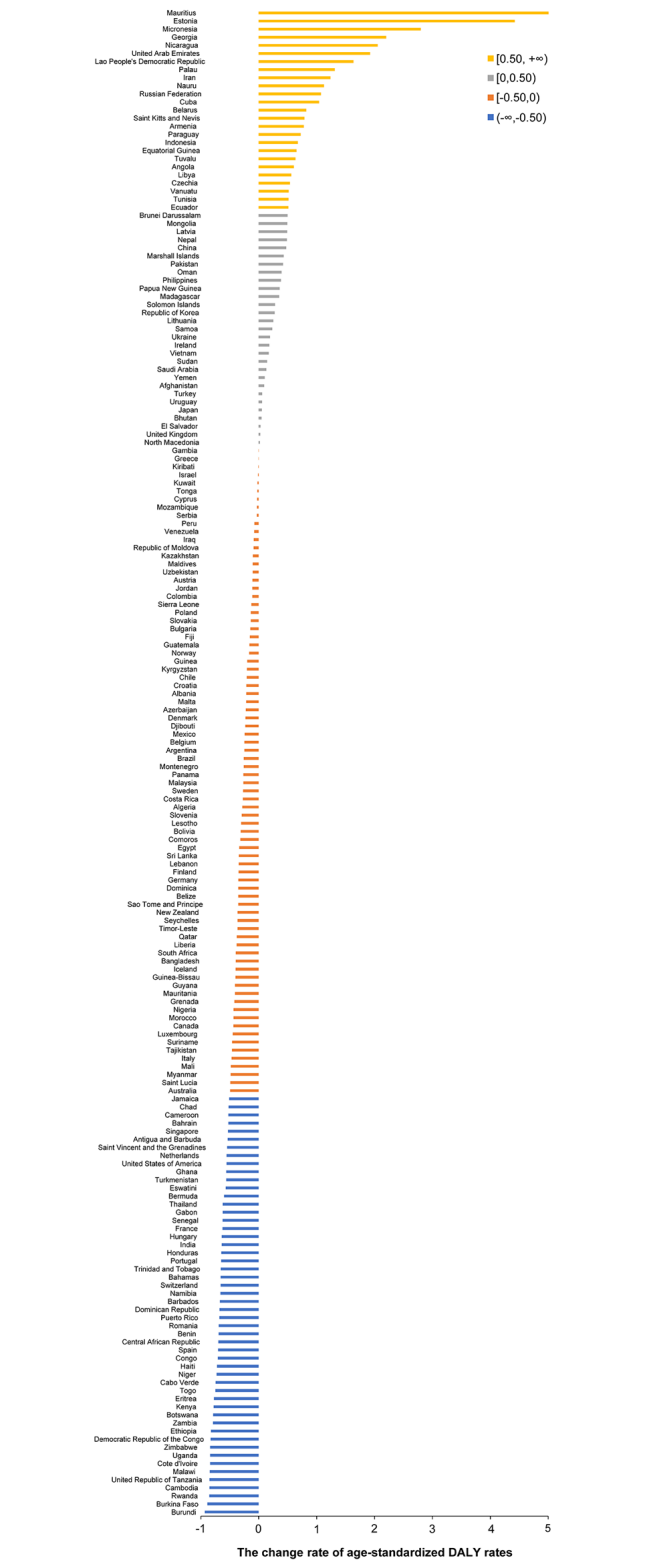
The change rate of age-standardized DALY rates for HIV/AIDS from 2000 to 2019. We calculated age-standardized DALY rates for HIV across 186 countries from 2000 to 2019. The change rate was calculated as: (age-standardized DALY rates in 2019 minus age-standardized DALY rates in 2000) / age-standardized DALY rates in 2000. Abbreviation: DALY, disability-adjusted life-year

Figure 1 shows the change rate of age-standardized DALY rates for HIV/AIDS from 2000 to 2019 among 186 countries and territories. Globally, the overall age-standardized DALY rates for HIV/AIDS decreased by 62.85% from 2000 to 2019, with annual changing rates showing a broad spatial variation among countries/territories. A decrease in age-standardized DALY rates for HIV/AIDS was observed in 132 (71%) of 186 countries/territories from 2000 to 2019, of which 52 (39%) achieved a decrease of more than 50%, and 27 (52%) of the 52 were from sub-Saharan Africa. The most prominent decrease was observed in Burundi, with a decrease of 93.23%. However, the remaining 54 countries/territories experienced an increase in age-standardized DALY rates for HIV/AIDS from 2000 to 2019, of which 25 (46%) countries/territories increased by more than 50%, and 10 (40%) of the 25 were from Europe and the Americas. Mauritius showed the highest (five-fold) increase (Fig. 1).

### Socioeconomic inequality in HIV/AIDS burden

**Fig. 2:**
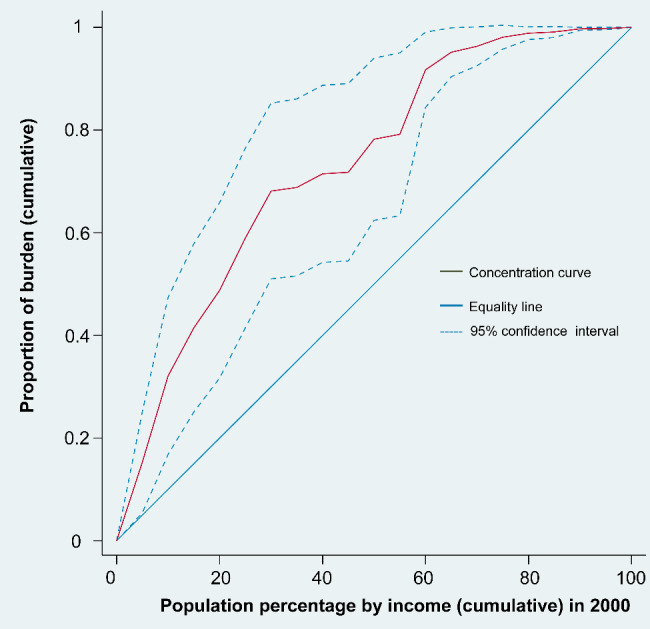
Concentration curve of age-standardized DALY rates for HIV/AIDS in 2000. The figure shows actual cumulative concentration curve for the change in cumulative proportion of HIV/AIDS burden with increase in cumulative percentage of countries ranked by income (GNI per capita) in 2000. Abbreviations: DALY, disability-adjusted life-year; GNI, gross national income

**Fig. 3:**
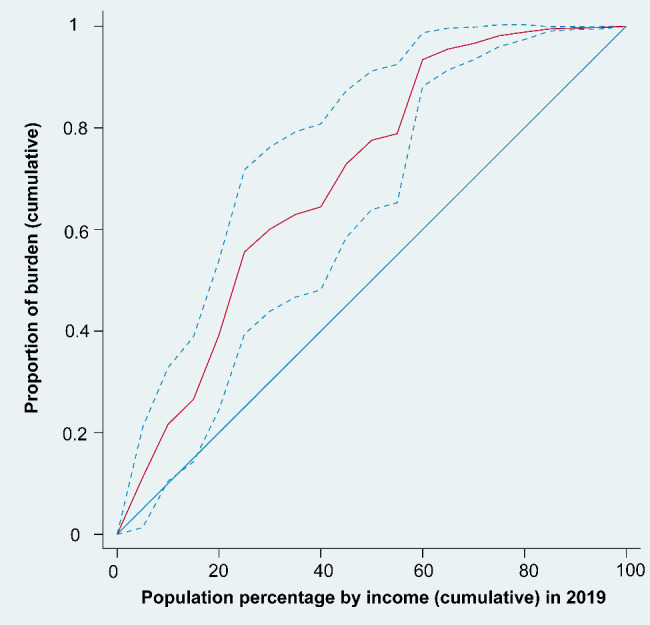
Concentration curve of age-standardized DALY rates for HIV/AIDS in 2019. The figure shows actual cumulative concentration curve for the change in cumulative proportion of HIV/AIDS burden with increase in cumulative percentage of countries ranked by income (GNI per capita) in 2019. Abbreviations: DALY, disability-adjusted life-year; GNI, gross national income

**Fig. 4:**
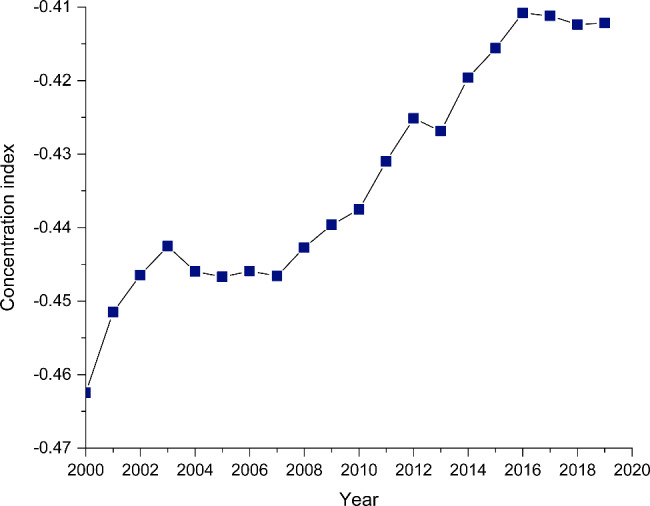
Concentration index of age-standardized DALY rates for HIV/AIDS from 2000 to 2019. Abbreviation: DALY, disability-adjusted life-year

Based on the concentration curve and index, the geographic distribution of age-standardized DALY rates due to HIV/AIDS demonstrates notable socioeconomic inequalities among the 186 countries/territories analyzed. The concentration curves were above the equality line from 2000 to 2019, with the 2000 curve furthest from the equality line, suggesting that the age-standardized DALY rates of HIV/AIDS were more endemic among poor countries/territories. In addition, the gap between the concentration curve and the equality line narrowed over time, with the CI rising from − 0.4625 (95% confidence interval − 0.6220 to -0.2629) in 2000 to -0.4122 (95% confidence interval − 0.6008 to -0.2235) in 2019. Despite this apparent reduction in socioeconomic-related inequality in HIV/AIDS burden, the current inequalities remain striking (Figs. 2, 3 and 4, Additional file: Fig.S1-18).

### Trends in socioeconomic inequality in HIV/AIDS burden

Globally, the CIs of age-standardized DALY rates for HIV/AIDS showed a pronounced increasing trend from 2000 to 2019 at an average increase of 0.6% (95% confidence interval 0.4 to 0.8) according to the joinpoint regression analysis. This result reveals that the disparities between the burden of HIV/AIDS sustained by poorer and better-off countries/territories are diminished slightly over time. A four-phase trend of changes in the CIs of age-standardized DALY rates for HIV/AIDS was observed over 2000–2019. The CIs increased significantly during the first and third interval, with an APC of 1.9% (95% confidence interval 0.7 to 3.1) from 2000 to 2002 and 0.9% (95% confidence interval 0.8 to 1.0) from 2007 to 2016. Conversely, the CIs showed a mild, insignificant reduction from 2002 to 2007, with an APC of -0.1% (95% confidence interval − 0.5 to 0.3), and no change from 2016 to 2019 (Table 1).

**Table 1:** Trends in concentration index of age-standardized DALY rates for HIV/AIDS from 2000–2019

Concentration index	Period	APC/AAPC (%)	95% confidence interval	P value
Trend 1	2000–2002	1.9*	0.7 to 3.1	0.007
Trend 2	2002–2007	-0.1	-0.5 to 0.3	0.448
Trend 3	2007–2016	0.9*	0.8 to 1.0	< 0.001
Trend 4	2016–2019	0	-0.6 to 0.6	0.966
Total	2000–2019	0.6*	0.4 to 0.8	< 0.001

APC = annual percentage change. AAPC = average annual percentage change

## Discussion

This study reports the socioeconomic inequalities, trends, and the changes in age-standardized DALY rates due to HIV/AIDS from 2000 to 2019. Our study found a substantial reduction in the global burden of HIV/AIDS over time, especially in LMICs. While the socioeconomic-related inequalities of HIV/AIDS burden reduced globally, an important finding of our study is that progress was skewed, with burden continuing to weigh more heavily in socioeconomically disadvantaged countries/territories. Socioeconomic inequalities of HIV/AIDS burden evolved through four phases, mirroring the uneven development of HIV prevention and control programs globally over the past two decades.

Our study confirmed a decrease in the age-standardized DALY rates of HIV/AIDS in most countries or territories worldwide (a remarkable achievement) with global trends indicating considerable progress in curbing the HIV/AIDS pandemic over the past two decades. This trend was consistent with the sustained declines in HIV/AIDS incidence and mortality globally since 2005 [7, 8, 11], and with health system reforms and effective control strategies undertaken to enhance access to and coverage of health care [25]. Within the Millennium Development Goals launched by the World Health Organization (WHO) in 2000, HIV/AIDS was established as a top global health priority and received substantial health resources [4, 5, 26]. A few major international organizations and foundations were expanded or initiated, with substantial funding provided annually to deliver HIV-related prevention, treatment, and financing protection services for regions struggling with a critical HIV epidemic [5]. The funding has been committed to controlling HIV transmission, including eliminating mother-to-child transmission, eliminating blood transmission, and controlling sexual and injecting drug transmission [3, 27]. In addition, the proactive advocacies of HIV education, healthy lifestyle behaviors, and mental support services have a lasting positive impact on reducing HIV incidence worldwide [27, 28]. The WHO had endorsed a series of resolutions to promote access to ARTin developing countries since 2000, aiming to reduce mortality and improve the quality of life of people living with HIV [29–31]. China initiated the free ART program in 2002 as a positive response to combat HIV/AIDS [32]. Sustained efforts have yielded solid progress, and an estimated 75% of people living with HIV globally were receiving ART in 2021 [2]. Prevention and ART were therefore of major significance in alleviating the global burden of HIV/AIDS.

Despite the fact that sub-Saharan Africa remains the epicenter of the HIV pandemic [8, 10], it is encouraging that HIV/AIDS burden has declined significantly over the past two decades. Much of the progress against HIV/AIDS in sub-Saharan African countries, including Burundi, Malawi, and Zimbabwe, may be attributable to increased health investments, improved healthcare systems, secondary and tertiary education, HIV self-testing programs, and adolescent male circumcision [33–36]. Furthermore, the “treat all” policy efficiently promoted the rapid initiation of ART in six sub-Saharan African countries from 2004 to 2018 [37]. However, the burden clearly increased in certain Eastern European and the American countries, concurring with a study by Govender et al. which indicated an increasing incidence of HIV/AIDS in these countries [38]. Reduction in health funding, men who have sex with men (MSM), and the spread of drug-resistant HIV strains are possible explanatory factors in this concerning finding [39–41].

We discovered that countries with a low GNI per capita were sharing an overwhelming burden of HIV/AIDS. In previous studies, the total burden of HIV/AIDS, including both incidence and mortality, was heavier in most LMICs than in high-income ones [7, 8, 11]. It is widely acknowledged that income is a core social determinant in human health [42], and its distributional inequalities and imbalances may exert a substantially adverse impact on health financing, access to healthcare, health insurance coverage, access to education, and health outcomes [43]. Owing to the absence of primary care infrastructures, persons living with HIV and at-risk populations from low-income countries such as those in sub-Saharan Africa failed to benefit from sufficient healthcare services, including timely HIV testing, mother-to-child program enrollment, and sustained ART treatment. This contrasts with improved quality of HIV care in high-income industrialized countries, such as the introduction of highly active antiretroviral treatment (HAART), which minimized viral replication levels via a combination of several efficacious antiretrovirals, leading to a striking decline in HIV incidence and mortality [3]. People living with HIV in high-income countries are primarily covered by social health insurance, Medicaid, private medical insurance, or a combination of these coverages [44], which provide free ART coverage and health counseling services to maintain continuity of patients’ healthcare [45]. However, a lack of universal health insurance coverage in most low-income countries accounts for the vulnerable health financing mechanisms [46], may result in people with HIV/AIDS declining treatment in response to persistent out-of-pocket expenditures, thus contributing to increased mortality. In addition, among countries with low socioeconomic status, limited education hampered the access to HIV care and diminished the readiness of the stigmatized populations to seek care [12]. Gender and racial inequalities were more prevalent in low-income countries, and women and people of color with HIV/AIDS were more exposed to denial of sexual and reproductive health rights, reducing their access to HIV-related medical care [47, 48]. HIV/AIDS is prone to a variety of complications if uncontrolled, resulting in greater costs of care [3]. Consistent with this, people living with HIV in low-income countries are more likely to undergo disease progression and deterioration due to the unaffordable cost of complications.

Our discovery of significantly reducing socioeconomic inequalities in HIV/AIDS burden between countries over time is encouraging, and mirrors the natural history of the HIV pandemic and the valid implementations of related strategies. This improving trend signifies the advances of the regional reduction: at lower socioeconomic class, HIV/AIDS burden is relatively heavy, and its decrease over time is greater. Consistent with this, the concentration curves tend towards equality (the diagonal) over time, indicating a narrowing gap in HIV/AIDS burden between low- and high-income countries, particularly in the periods 2000–2002 and 2007–2016, though the trend was modest. The accomplishments of inequality reduction in both periods are attributable primarily to the continuing support of international assistance organization [5]. However, our study found a slight, insignificant rise in inequality of the global HIV/AIDS burden in the period 2002–2007. One possible explanation for this finding is that developed countries were more capable of tackling the HIV pandemic and delivering an early response based on an already robust health system. We also found that the global campaign to tackle the unequal burden of HIV/AIDS from 2016 to 2019 was stagnant, a cautionary signal for global health.

Our analysis of cross-national socioeconomic inequality of HIV/AIDS burden is beneficial in illustrating global patterns of HIV inequality and refining the current control strategies. Sustained efforts to combat the HIV pandemic over the past two decades have resulted in progress toward reducing the overall global burden of HIV/AIDS. However, slow progress was made in addressing the socioeconomic-related inequalities in the HIV/AIDS burden and related morbidity and mortality, and no substantial strides in HIV-related intervention strategies have been made in recent years, implying that many countries are unlikely to realize UNAIDS 2030 morbidity and mortality targets [8]. With increased life expectancy, the number of people living with HIV is growing, and HIV carriers are at high risk of COVID-19 complications [49], which continue to place an increasing burden on health systems, particularly in low-income countries. Further expansion of health assistance for LMICs is urgently warranted to accelerate the pace of progress. Early diagnosis and intervention are regarded as the most cost-effective strategies [50], especially for regions with restricted health resources. These approaches should be combined with enhanced education, disease awareness and protection, in an effort to avert or reduce the adverse health consequences of HIV, and this demands collaborative cross-sectoral actions targeting the social determinants of health. Thus, as a protective HIV vaccine is not available, further consolidation and upscaled HIV surveillance and testing, risk consultation, PrEP and ART access, and other support programs are crucial. From a high-risk population perspective, there is a compelling need for the HIV/AIDS response to focus on women in regions with high-endemic poverty, who are exposed to the most profound inequalities in HIV/AIDS care, including stigma, denial of treatment, and violence [6]. Targeted interventions should surpass HIV suppression and provide full consideration of the social, economic, legal, psychological and emotional demands in combating the HIV pandemic. Drawing on the evidence and experience obtained over the past two decades, a multi-pronged, multi-tiered strategy is needed to minimize HIV inequality across countries and to end the HIV pandemic.

### Strengths and limitations

A strength of the current study is the comprehensive population-based assessment and comparison of trends in socioeconomic inequalities of HIV/AIDS burden worldwide based on nations’ income levels, which adds to the evidence base for the development and implementation of future HIV inequality-eradication strategies. Several limitations should be considered. First, as with other GBD analyses, the accuracy of estimates in our study depends on the quality and quantity of data sources. These were limited by detection techniques, incomplete case-reports, and data collection and encoding methods used in different countries. Second, the absence of epidemiological surveys in certain regions may result in a hidden incidence, particularly in low-income countries, which means that inequality of HIV/AIDS burden is potentially underestimated. Finally, our study is cross-national, which may introduce bias due to a lack of knowledge of disparities that exist between districts within countries.

## Conclusion

Globally, the overall burden of HIV/AIDS has decreased substantially over the past 20 years, especially in sub-Saharan African countries. Socioeconomic inequality in global burden of HIV/AIDS has improved over this period, but low-income countries continue to share the major burden of HIV/AIDS worldwide. Adherence to prevention-oriented strategies and enhanced financial assistance to economically vulnerable nations are critical in eliminating HIV inequality.

## Electronic supplementary material

Below is the link to the electronic supplementary material.

Supplementary Material 1

## Data Availability

Data are publicly available. The datasets generated and analysed during the current study are available in the Global Health Data Exchange GBD 2019 data resources (https://ghdx.healthdata.org/gbd-2019).

## Abbreviations

GBD: The Global Burden of Disease, Injuries, and Risk Factors Study
DALY: Disability-adjusted life-year
GNI: Gross national income
CI: Concentration index
HIV: Human immunodeficiency virus
AIDS: Acquired immunodeficiency syndrome
LMICs: Low- and middle-income countries
UNAIDS: United Nations Programme on HIV/AIDS
ART: Antiretroviral treatment
SDGs: Sustainable development goals
COVID-19: Corona Virus Disease 2019
YLLs: Years of life lost
YLDs: Years lived with disability
APC: Annual percentage change
AAPC: Average annual percentage change
WHO: World Health Organization
PeEP: Pre-exposure prophylaxis
MSM: Men who have sex with men
HAART: Highly active antiretroviral treatment
IHME: Institute for Health Metrics and Evaluation
