# Supplementary material for: Temporal trends in inequalities of the burden of HIV/AIDS across 186 countries and territories
Source: BMC Public Health. 2023 May 26;23:981. doi: 10.1186/s12889-023-15873-8 (PMC10215035; doi:10.1186/s12889-023-15873-8)

**Additional file: Fig.S1-18: Concentration curves of age-standardized DALY rates for HIV/AIDS from 2001 to 2018**

**Figure S1. Concentration curve of age-standardized DALY rates for HIV/AIDS in 2001**


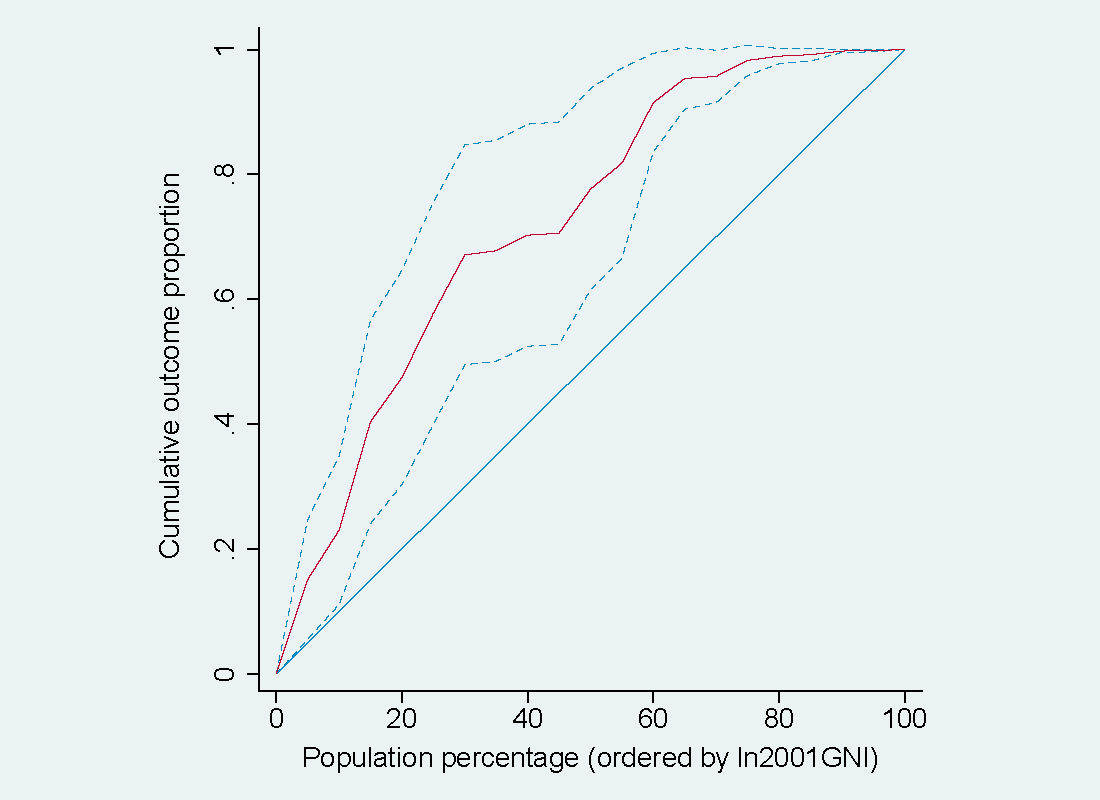
 **Figure S2. Concentration curve of age-standardized DALY rates for HIV/AIDS in 2002**


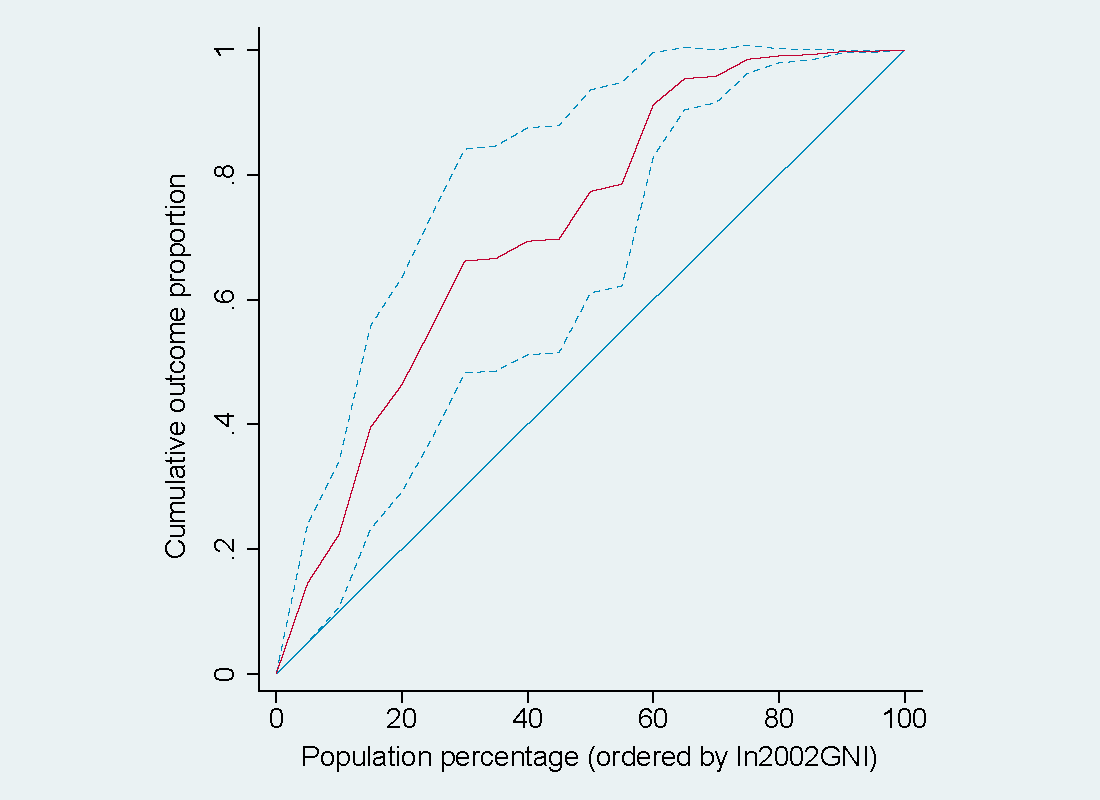


**Figure S3. Concentration curve of age-standardized DALY rates for HIV/AIDS in 2003**


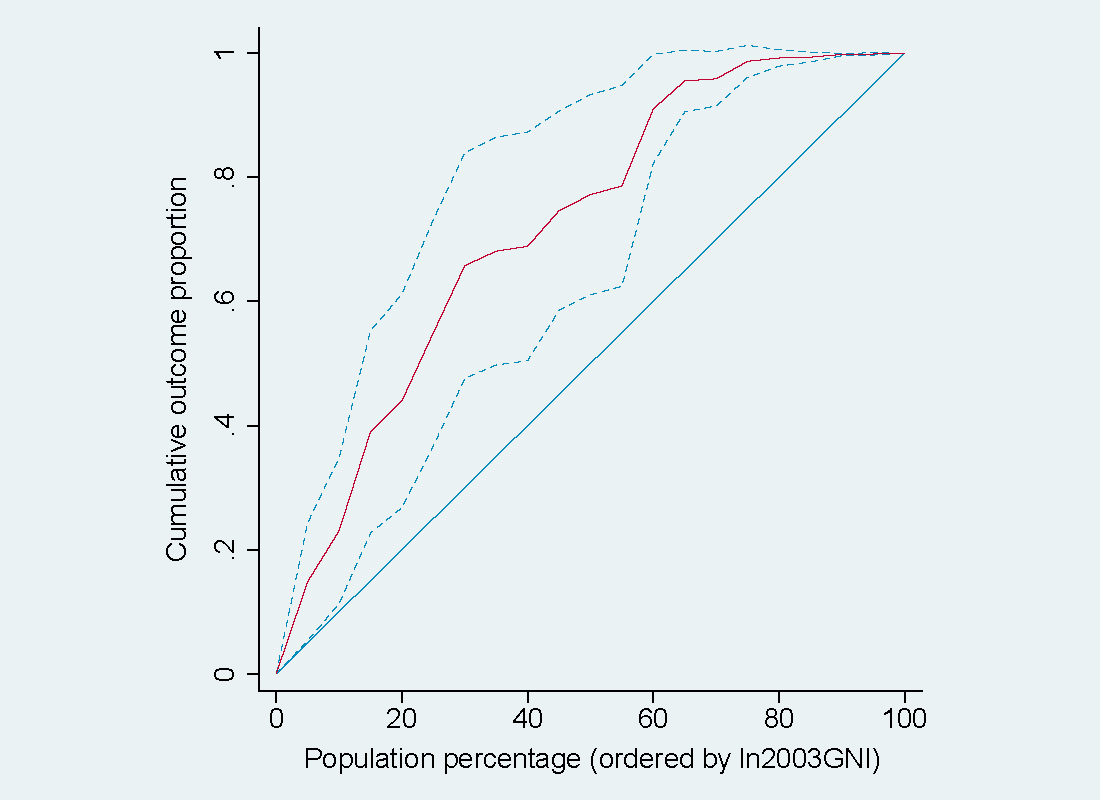
 **Figure S4. Concentration curve of age-standardized DALY rates for HIV/AIDS in 2004**


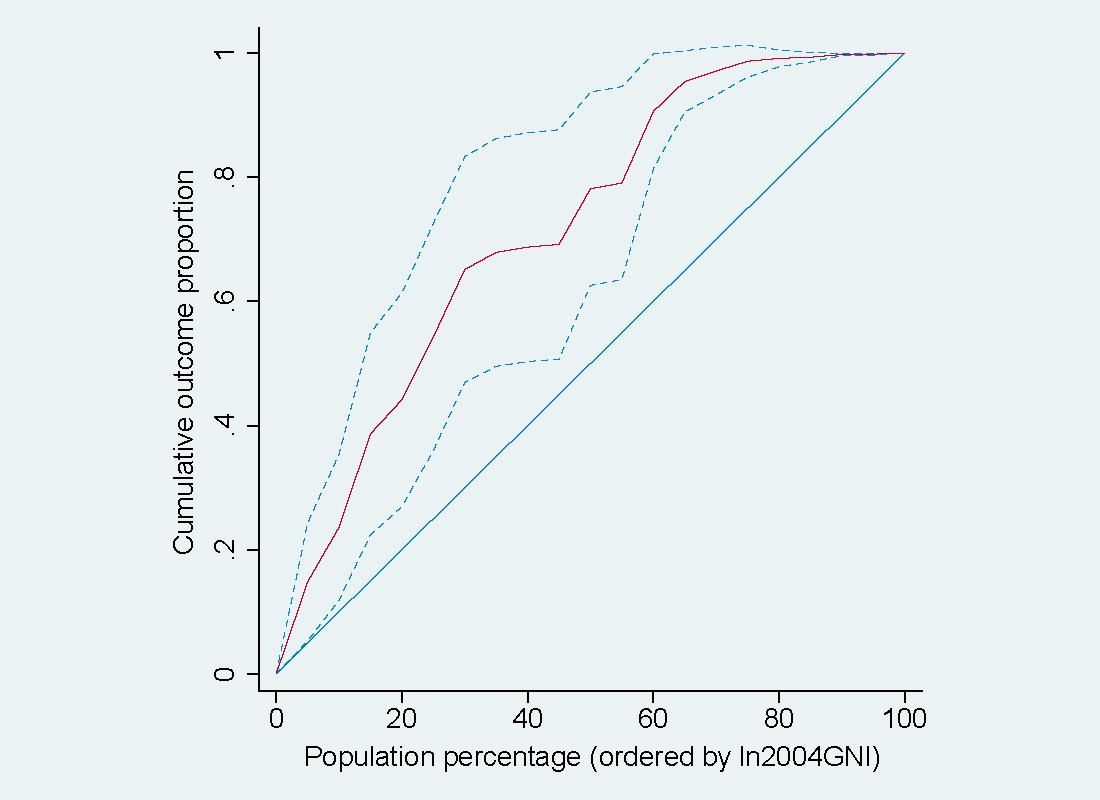


**Figure S5. Concentration curve of age-standardized DALY rates for HIV/AIDS in 2005**


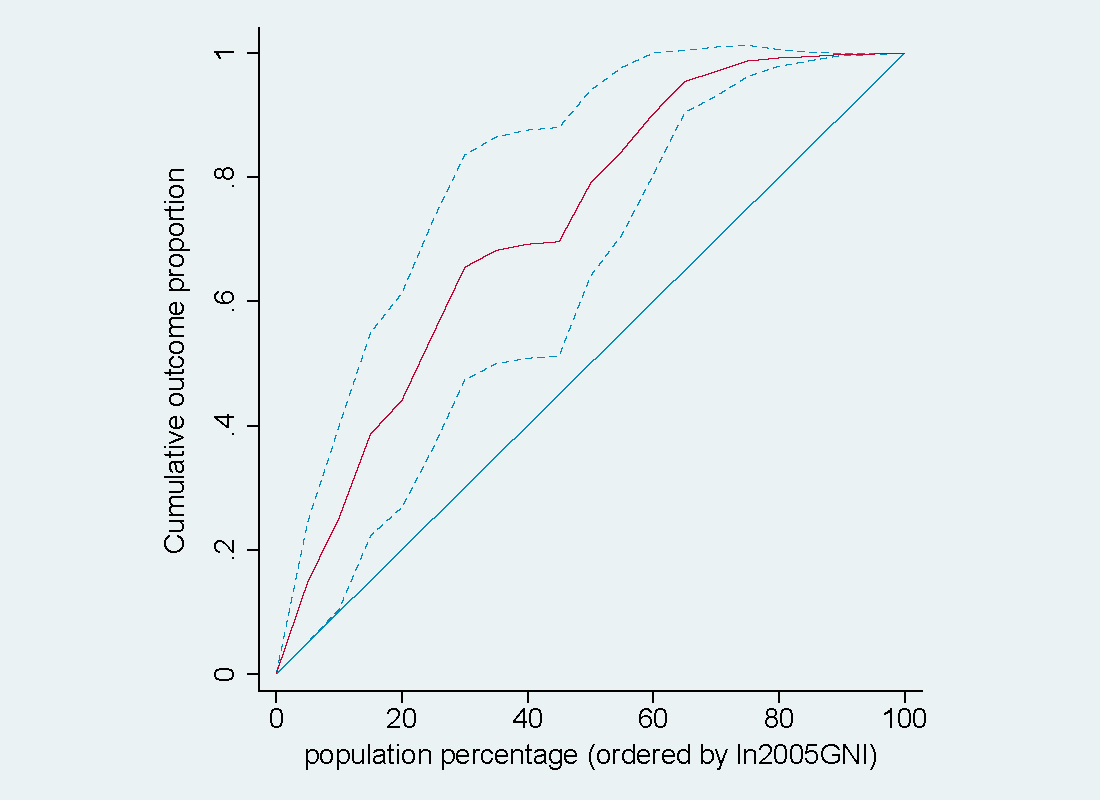
 **Figure S6. Concentration curve of age-standardized DALY rates for HIV/AIDS in 2006**


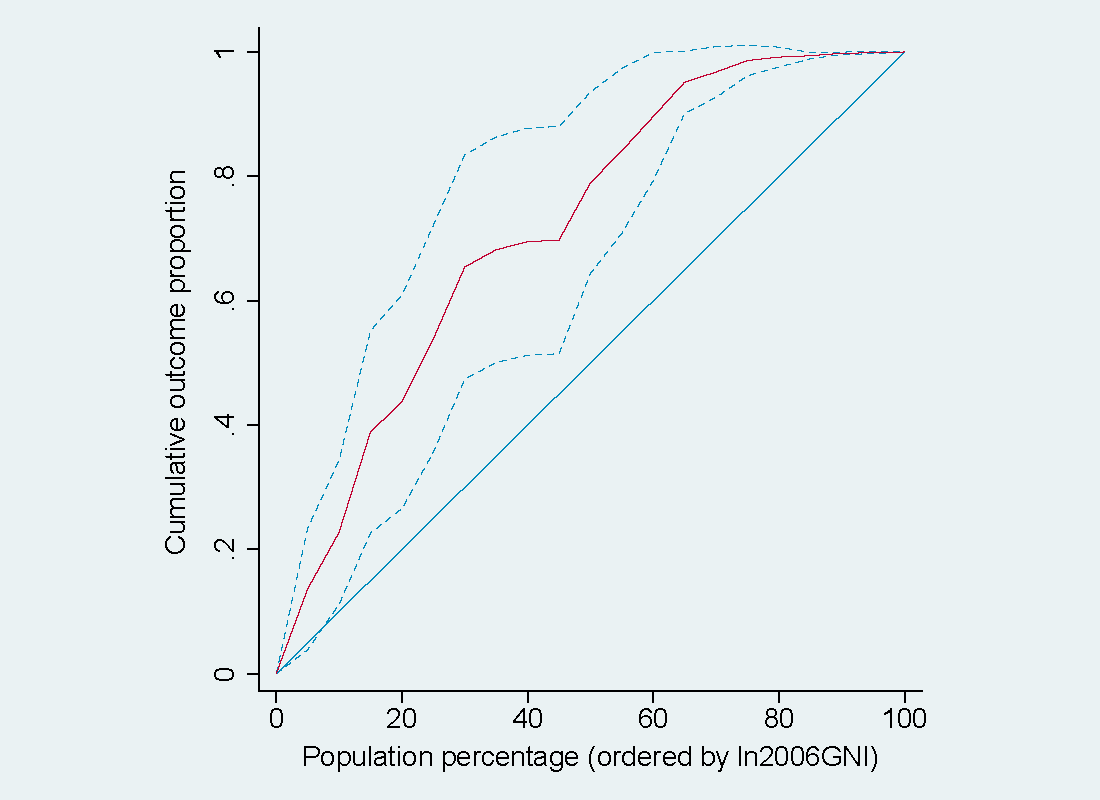


**Figure S7. Concentration curve of age-standardized DALY rates for HIV/AIDS in 2007**


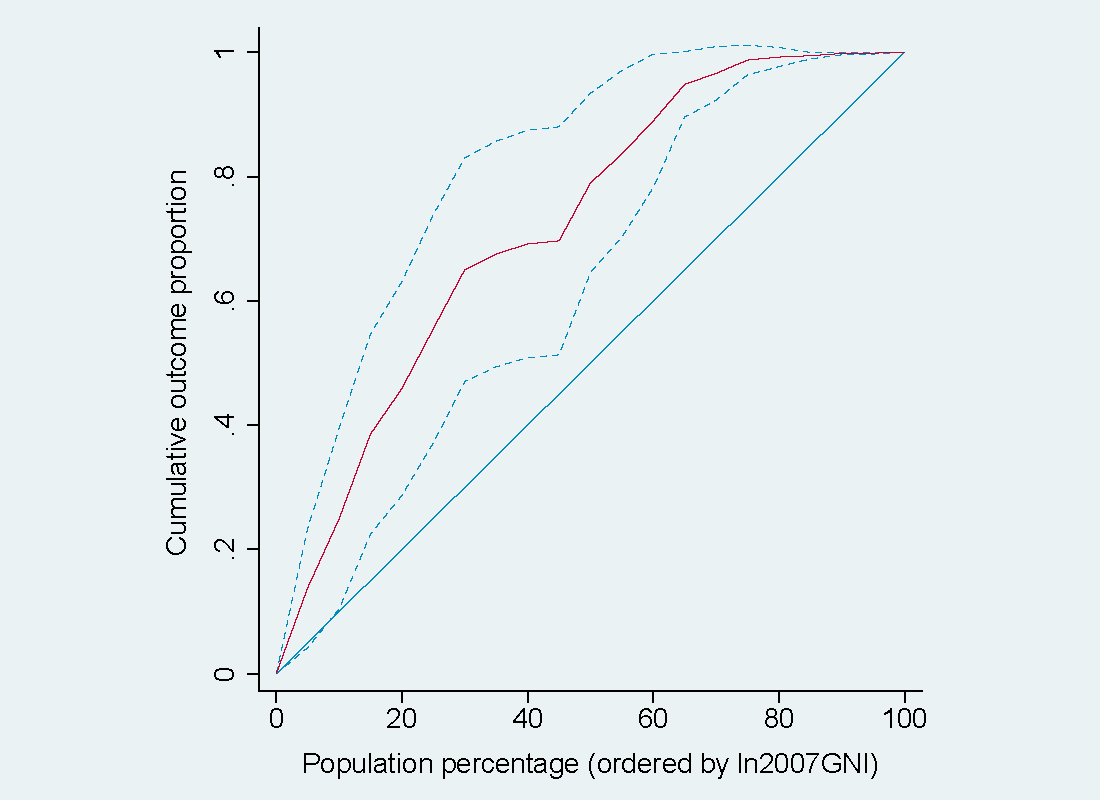
 **Figure S8. Concentration curve of age-standardized DALY rates for HIV/AIDS in 2008**


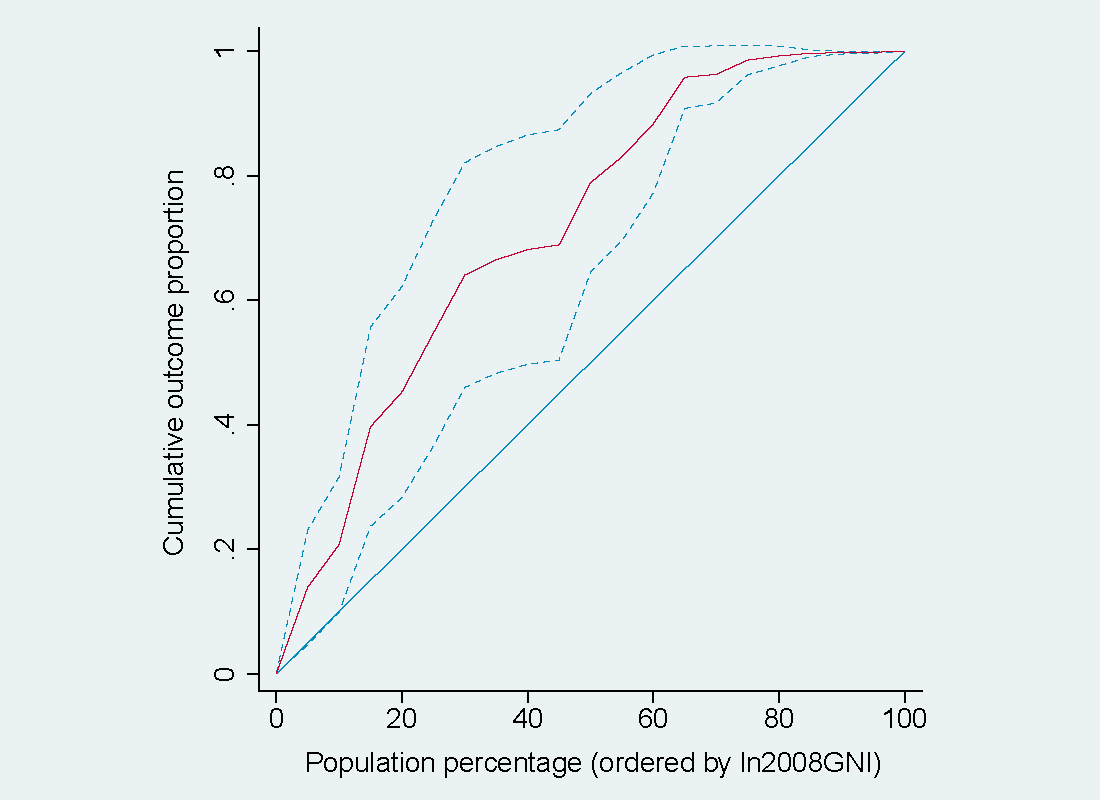


**Figure S9. Concentration curve of age-standardized DALY rates for HIV/AIDS in 2009**


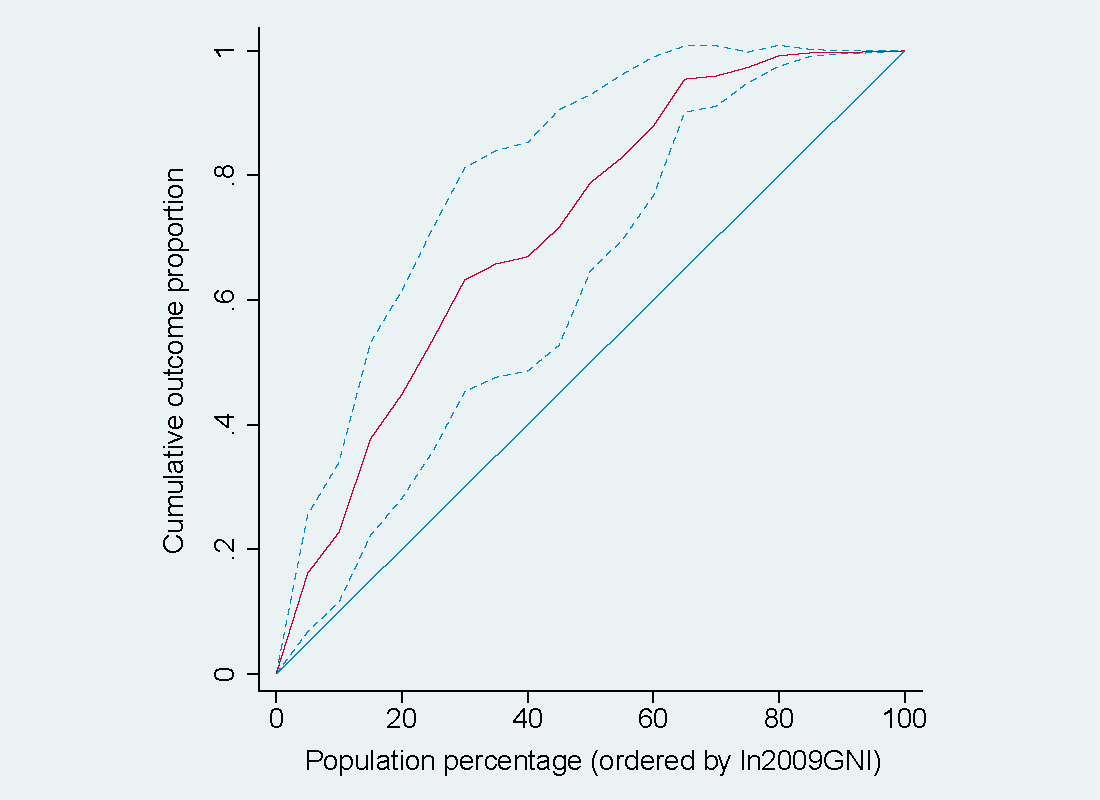
 **Figure S10. Concentration curve of age-standardized DALY rates for HIV/AIDS in 2010**


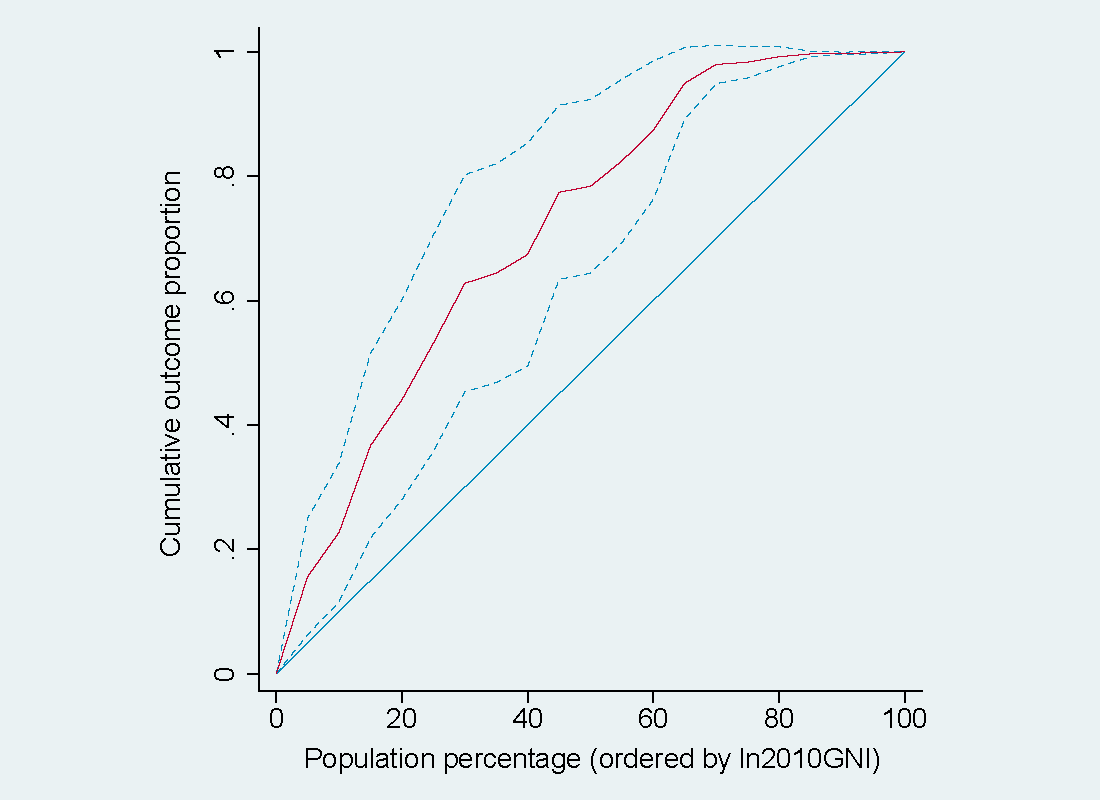


**Figure S11. Concentration curve of age-standardized DALY rates for HIV/AIDS in 2011**
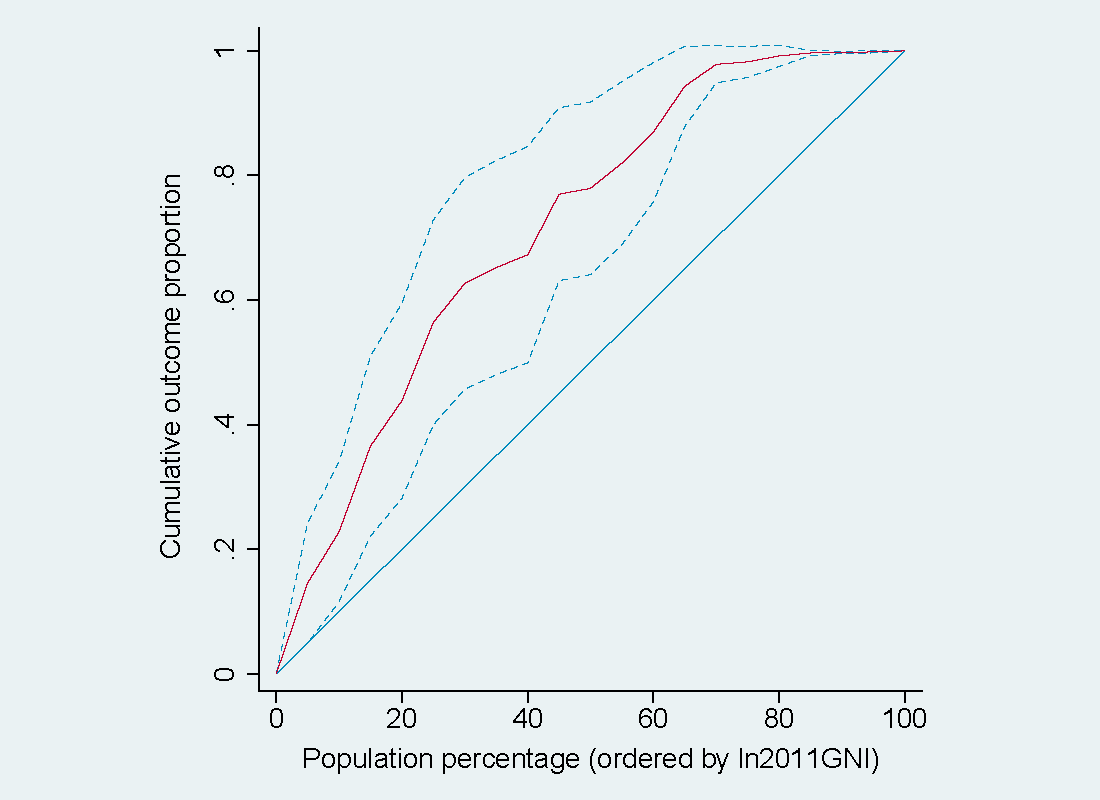
 **Figure S12. Concentration curve of age-standardized DALY rates for HIV/AIDS in 2012**
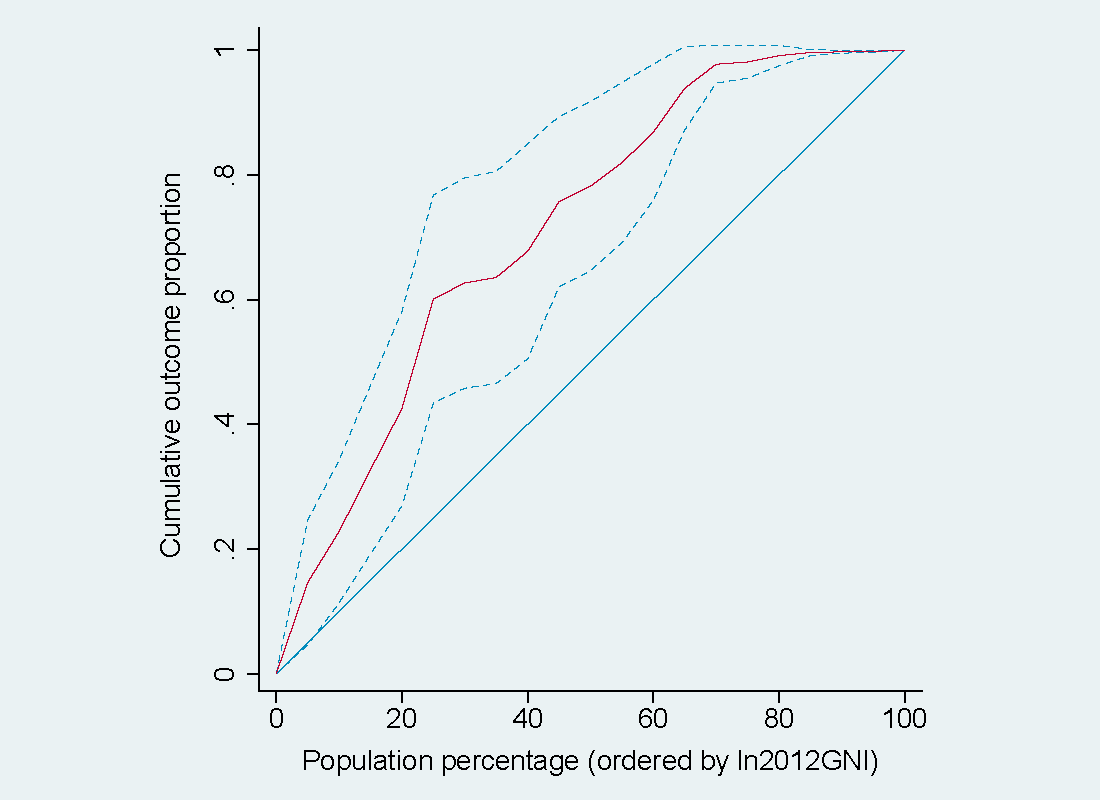


**Figure S13. Concentration curve of age-standardized DALY rates for HIV/AIDS in 2013**
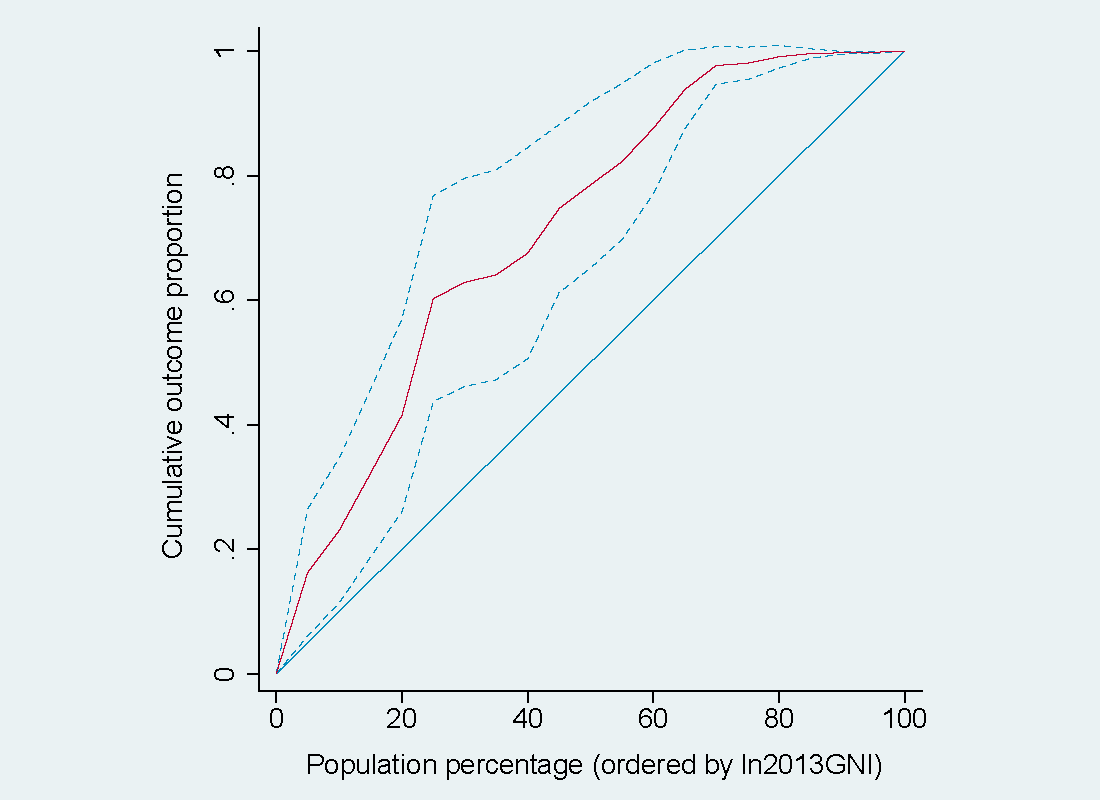
 **Figure S14. Concentration curve of age-standardized DALY rates for HIV/AIDS in 2014**
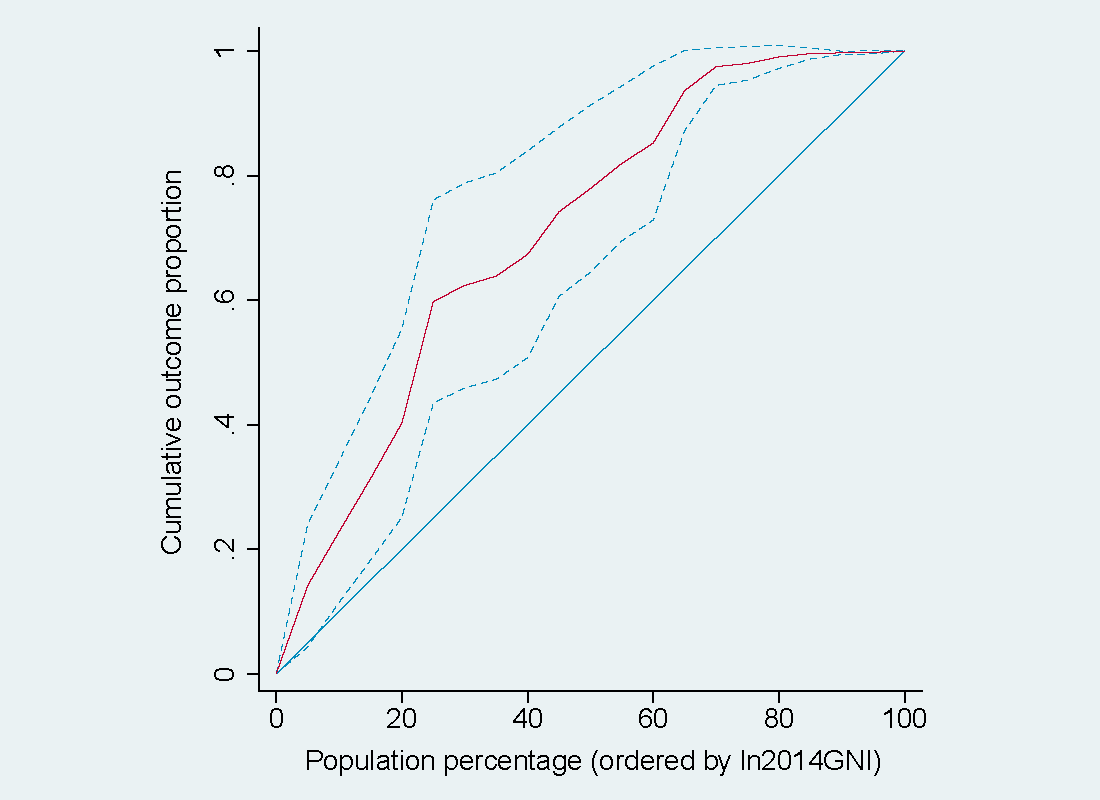


**Figure S15. Concentration curve of age-standardized DALY rates for HIV/AIDS in 2015**
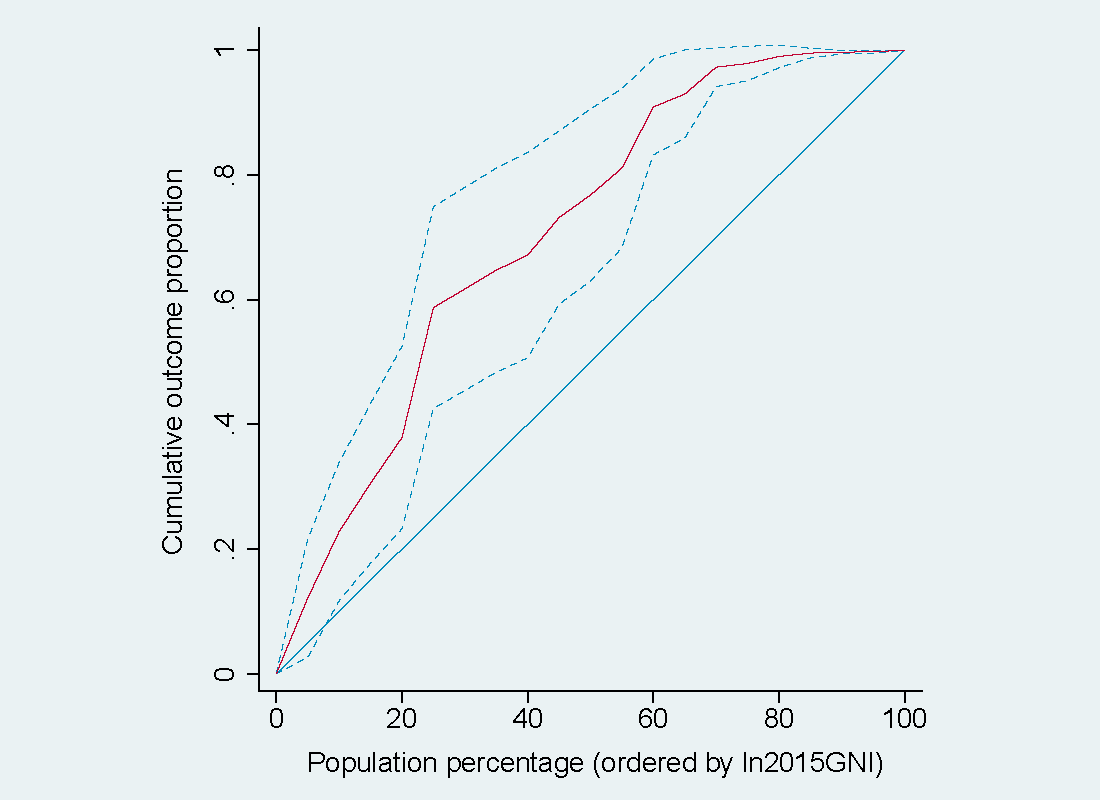
 **Figure S16. Concentration curve of age-standardized DALY rates for HIV/AIDS in 2016**
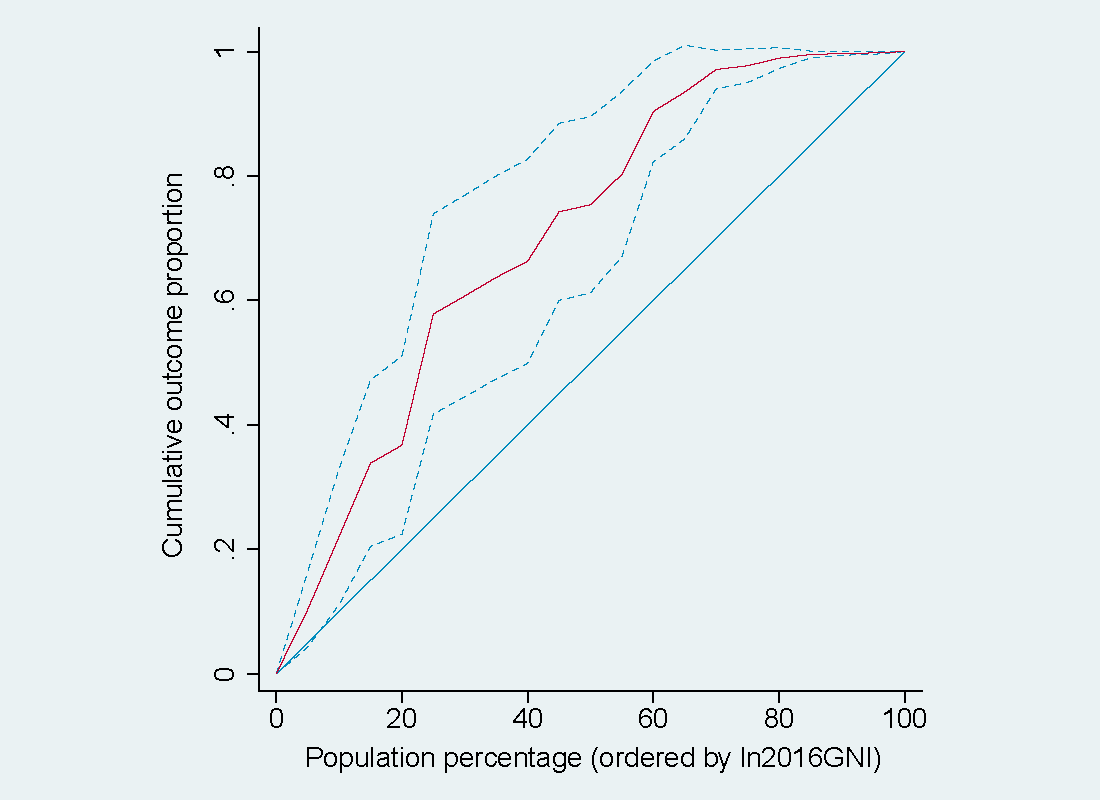


**Figure S17. Concentration curve of age-standardized DALY rates for HIV/AIDS in 2017**
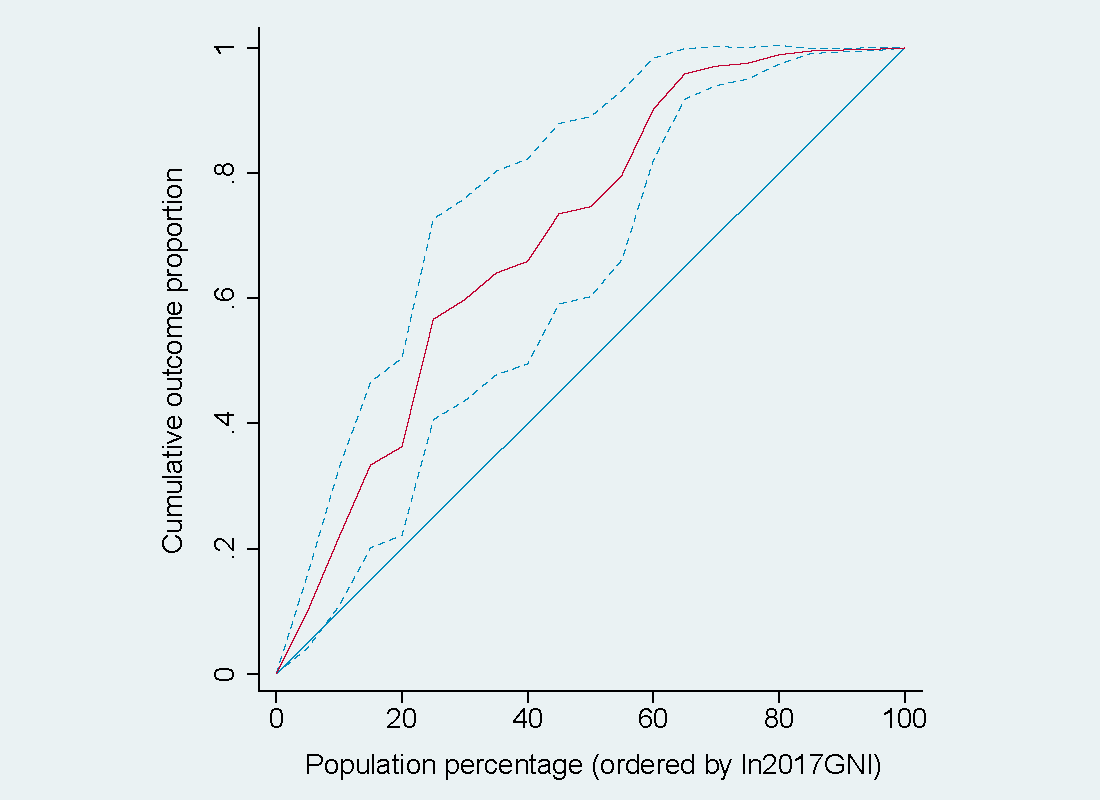
 **Figure S18. Concentration curve of age-standardized DALY rates for HIV/AIDS in 2018**
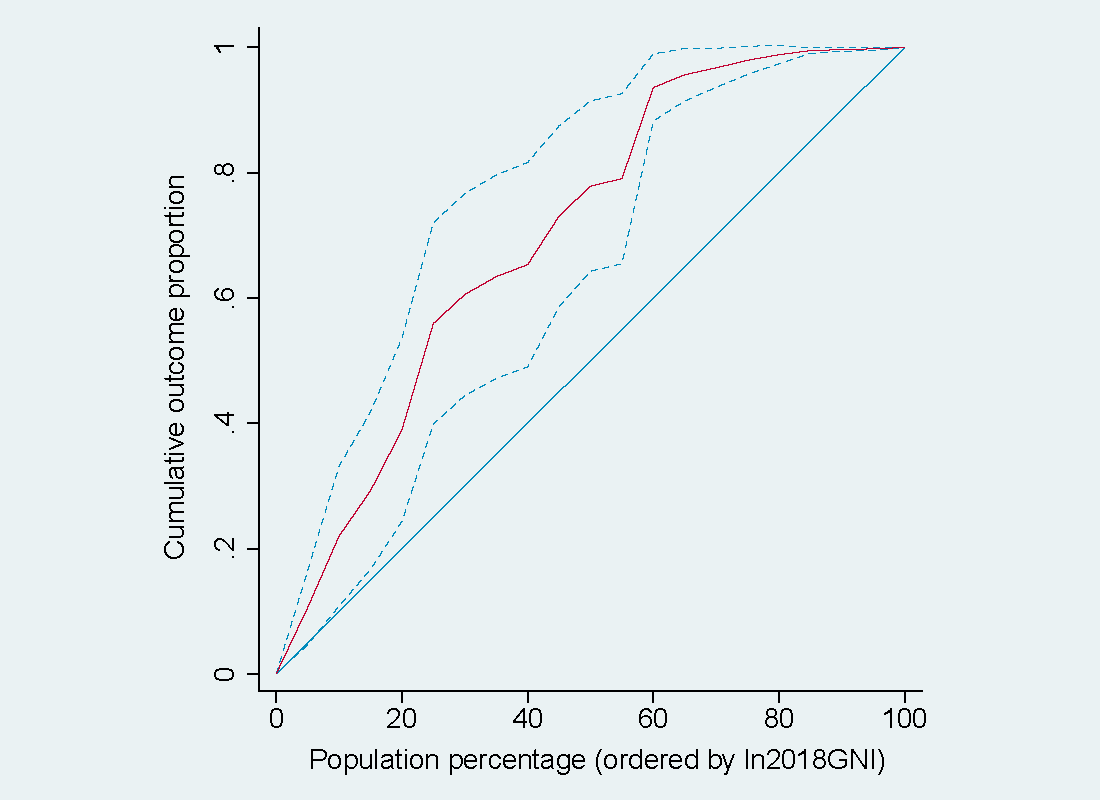

Supplement: Supplementary file 1 — Supplementary Material 1 [file 12889_2023_15873_MOESM1_ESM.docx]
